# Personalized therapy: can it tame the COVID-19 monster?

**DOI:** 10.2217/pme-2021-0077

**Published:** 2021-10-15

**Authors:** Mohd Arish, Farha Naz

**Affiliations:** 1 ^1^JH-Institute of Molecular Medicine, Jamia Hamdard, New Delhi, India; 2 ^2^Department of Immunology, Division of Pulmonary & Critical Care Medicine, Mayo Clinic, Rochester NY 55902, USA; 3 ^3^Centre for Interdisciplinary Research in Basic Sciences (CIRBSc), Jamia Millia Islamia, New Delhi, India

**Keywords:** adverse effects, antivirals, biomarkers, COVID-19, drug repurposing, drugs, drug toxicity, personalized medicine, precision therapy, SARS-CoV-2

## Abstract

SARS-CoV-2, a recently emerged zoonotic virus, has resulted in unstoppable high morbidity and mortality rates worldwide. However, due to a limited knowledge of the dynamics of the SARS-CoV-2 infection, it has been observed that the current COVID-2019 therapy has led to some clinical repercussions. We discuss the adverse effects of drugs for COVID-2019 primarily based on some clinical trials. As therapeutic efficacy and toxicity of therapy may vary due to different, genetic determinants, sex, age and the ethnic background of test subjects, hence biomarker-based personalized therapy could be more appropriate. We will share our thoughts on the current landscape of personalized therapy as a roadmap to fight against SARS-CoV-2 or another emerging pathogen.

SARS-CoV-2 has become a global threat affecting more than 200 countries leading to more than 160 million confirmed cases and 3 million deaths worldwide, as of June 2021 [1]. Already declared as a global public health emergency by the WHO, the ongoing COVID-19 pandemic has driven the development of effective therapeutic agents, and as a result repurposed drugs are currently being tested against COVID-19. Although vaccination drives all over the world have now halted the infection spread, some breakthrough infection is reported in fully vaccinated individuals [2]. Hence, in addition to vaccines, there is an unmet need for a potential treatment for COVID-19.

Following the COVID-19 outbreak, researchers and clinicians are racing toward finding a drug that can exert antiviral activity with minor adverse effects and should be of low cost. Considering that SARS-CoV-2 is a recently emerged virus, the only viable options available, apart from vaccines, are repurposed drugs. Clinical trials are being conducted based on our previous experiences with these antiviral drugs against highly pathogenic RNA viruses such as HIV, Ebola, influenza, etc. In addition to antiviral drugs, several clinical studies have also come to the common conclusion that COVID-19 results in hyper-secretion of cytokines and chemokines [3], and hence immuno-modulatory US FDA-approved drugs may also help to dampen the aberrant inflammatory immune response. Overall, it is suggested that combinational therapy with antiviral and anti-inflammatory drugs may be able to mitigate the current COVID-19 pandemic [4,5].

Here we have focused on some of the clinical complications reported in the test subjects that were on COVID-19 therapy (Table 1). To this end, we further suggest some factors behind the variable response to COVID-19 therapy, which primarily consists of polymorphism in gene-regulating drug metabolism and bioavailability. Cumulatively, a proper understanding of the strength and weaknesses of a drug may be helpful for clinicians in the proper management of the COVID-19 pandemic but, more importantly, it suggests that we must revisit our healthcare system. As there are differential responses to current therapy, a more precise approach could be needed in the time when we are still learning about COVID-19. At last, we share some thoughts on personalized medicine and why it should be adopted in our healthcare system.

**Table 1.** Drugs in clinical trials with the mode of action and associated adverse effects.

Drugs	Mode of action	Complications	Ref.
Chloroquine/hydrochloroquine	Impaired binding of SARS-CoV-2 spikes proteins with angiotensin-converting enzyme-2	Nausea, vomiting, blurred vision, rashes, headache	[6–8]
Remdesivir	Inhibits RNA virus replication	ARDS, multi-organ dysfunction, cardiopulmonary failure, secondary infection, recurrence of COVID-19	[9,10]
Lopinavir/ritonavir	HIV-1 protease inhibitor	Nausea, vomiting, diarrhea, ineffective to clear viral load	[11]
Favipiravir	Inhibits viral RNA polymerase	Increased serum uric acid	[12]
Corticosteroids	Mitigate hyper-immune response	Delays viral clearance, associated with mortality	[13–15]

ARDS: Acute respiratory distress syndrome.

## Clinical complications of drugs in trials

Based on the currently published clinical trials we tried to highlight the efficacy and side effects of drugs that are among the top contenders for COVID-19 therapy.

### Chloroquine/hydroxychloroquine

Chloroquine, an FDA-approved antimalarial drug, has shown inhibition of SARS-CoV-2 entry in Vero E6 cells line, as a result of impaired binding of SARS-CoV-2 spike proteins with angiotensin-converting enzyme-2 [16], that results in containment of the infection. Recently, chloroquine and its derivatives are showing promising results in the newly emerged pathogen, SARS-CoV-2 [6,17,18]. In addition to blocking the cellular uptake of SARS-CoV-2, chloroquine could also be beneficial in dampening the SARS-CoV-2 induced cytokine storm in infected patients. As initial clinical data showed the therapeutic potential of chloroquine in COVID-19 but still it was insufficient to draw any conclusion due to the fewer number of study subjects involved and no long-term follow-up in treated patients.

As hydroxychloroquine (HCQ) is a less toxic metabolite of chloroquine with potent *in vitro* anti-SARS-CoV-2 activity, it is currently being tested in various clinical trials. A randomized control trial enrolling 62 COVID-19 patients, 31 in the HCQ group and 31 in the control group were done to assess the efficacy of HCQ. The study, published as a preprint, reveals that the subject in the HCQ group showed an improvement in body temperature and a reduction of cough emission time. This study, with 62 randomized controls, showed that out of 31 subjects that were receiving HCQ only two developed minor symptoms such as rashes and headache. In addition, 67.7% of the patients on the HCQ therapy results in significant improvement of pneumonia [7]. A prospective study with 80 test subjects who were given 200 mg of oral HCQ sulfate combined with 500 mg azithromycin showed that only seven subjects reflected minor adverse effects. Two of the test subjects reported nausea and vomiting, while four of the subjects reported diarrhea and one test subject also reported blurred vision [8]. However, due to the small number of test subjects in these clinical trials, the adverse effect of HCQ therapy cannot be guaranteed. Recently, an observational study published in the New England Journal of Medicine stated that the HCQ treatment does not lower/exacerbates the disease. The study further discourages the use of HCQ outside randomized clinical trials [19]. At last, it is suggested that HCQ treatment may not be suitable for severe COVID-19 patients.

### Remdesivir

Remdesivir is a nucleotide analog and has potent antiviral activity against RNA viruses, such as Coronaviridae and Flaviviridae [20]. Remdesivir acts as an RNA-dependent RNA polymerase inhibitor and hence hinders the RNA replication process of the virus [21]. Earlier developed to treat Ebola infection, remdesivir is now repurposed against SARS-CoV-2 due to its effective *in vitro* antiviral activity against SARS-CoV-2 [6].

Intravenous treatment of remdesivir in 53 patients resulted in the improved clinical outcome in terms of oxygen support for 36 patients [9]. Out of 53 patients, 32 patients showed moderate adverse effects such as an increase in hepatic enzymes, diarrhea and rashes [9]. The most serious complication arises in 12 patients, including multiple-organ dysfunction syndrome, septic shock and kidney injury hypotension developed as a common adverse effect. Seven of the total patients under remdesivir died after completion of the treatment [9]. However, the lack of randomized control and small group size in the above study is a major limitation of the trial. In a more recent randomized clinical trial, remdesivir could not significantly reduce viral RNA load in the upper respiratory tract or sputum. This study includes 158 patients in the remdesivir group and 79 in the placebo group. Minor adverse effects such as hypoalbuminemia, hypokalaemia, anemia and thrombocytopenia were reported in 66% of the patients enrolled in remdesivir group. In addition, severe adverse effects including acute respiratory distress syndrome (ARDS), cardiopulmonary failure, secondary infection and recurrence of COVID-19, were also reported in 18% of the patients that led to discontinuation of remdesivir administration [10]. Although remdesivir is used as an emergency treatment for severe COVID-19 and has shown favorable outcomes, more large-scale clinical trials are suggested [22].

### Lopinavir/ritonavir

Lopinavir/ritonavir, an FDA-approved drug, is an HIV-1 protease inhibitor and thus prevents the formation of new virus particles. Large numbers of clinical trials have advocated lopinavir/ritonavir therapy as an effective therapy for HIV [23].

A randomized control study comprised of 199 patients (99 under lopinavir/ritonavir and 100 under standard treatment) with confirmed SARS-COV-2 infection was conducted to test the therapeutic efficacy of lopinavir/ritonavir. The study did not find any difference in the clearance of the viral load, nevertheless, the drug was safer than the standard therapy [11]. However, the study showed that the treatment of lopinavir/ritonavir in 46 patients results in gastrointestinal adverse effects including nausea, vomiting and diarrhea [11]. These side effects hence discourage the prolonged use of lopinavir/ritonavir in patients to overcome COVID-19. In a later clinical study, it was concluded that a lopinavir/ritonavir was not able to clear viral loads as significantly as arbidol, and at the end of the 14-day treatment, 44.1% of the patients were still detected with SARS-CoV-2 RNA [24]. As the study includes only 50 patients (34 in the lopinavir/ritonavir group and 16 in the arbidol group), the authors strongly advocate studying the effect of lopinavir/ritonavir in a large sample size to clarify its anti-SARS-CoV-2 activity.

### Favipiravir

Favipiravir inhibits influenza viral RNA polymerase by interacting with viral RNA polymerase [25]. Favipiravir is converted into a pro-drug, phospho-ribosylated form, which is taken up by viral RNA-dependent RNA polymerase as a substrate. Favipiravir was previously suggested to be used against several emerging RNA viruses such as Ebola and Lassa virus [25].

A study that includes 236 confirmed COVID-19 cases, was randomized into two groups, one under favipiravir treatment and the other under arbidol treatment. The favipiravir treated group did not show significant improvement in the recovery rate than the arbidol treated group. In addition, favipiravir resulted in more antiviral associated adverse effects in 37 patients [12]. Interestingly, favipiravir was able to improve the clinical recovery of patients with moderate COVID-19, however, due to the lack of placebo control, the efficacy of favipiravir could not be determined. A clinical trial has been recently approved to check the safety of favipiravir in moderate COVID-19 infections (clinical trial identifier no: NCT04336904). As per the study design, the investigators are planning to perform a double-blind, placebo-controlled clinical study, which will further expand the possibility of this drug being used against COVID-19.

### Corticosteroids

In most cases of COVID-19, increased levels of pro-inflammatory cytokines such as IL-2, TNF-α, IL-1β, IFN-γ and IL-6 and chemokines such as monocyte chemo-attractant protein-1 and macrophage inflammatory protein-1α were observed [26,27]. Based on these clinical features, these patients were on corticosteroid therapy, in addition to other medications, to subside the SARS-CoV-2 induced cytokine storm [26]. However, clinical data represents that corticosteroid therapy must not be given to SARS-CoV-19 infected patients as it results in delayed viral clearance [13].

Corticosteroids are generally recommended to COVID-19 patients to subside the hyper-immune response in the form of ARDS. However, there is still controversy in claiming that corticosteroids are beneficial to COVID-19 patients as some of the studies reported associated mortality with the treatment [14], and hence careful monitoring is required. More importantly, it is believed that optimum doses of corticosteroids must be administered during early infection as the corticosteroids can be more helpful in dampening the inflammation rather than clearing the viral load [28]. A study on 31 COVID-19 positive patients, without ARDS, out of which 11 patients received 40 mg methylprednisolone showed that the corticosteroid treatment does not influence viral clearance [15]. A meta-analysis report from 15 studies, including about 5300 patients, concluded that corticosteroid treatment was associated with mortality [29]. Hence, these reports cumulatively suggest the cautious use of corticosteroids for the treatment of COVID-19.

### Convalescent plasma

In convalescent plasma (CP) transfusion, the blood is collected from recovered COVID-19 patients and screened for SARS-CoV-2 specific neutralizing antibodies. The serum containing high titers of neutralizing antibodies is then transfused to the infected individual [30]. The antibodies then bind to the virus thus blocking their entry to host cells or can also mediate antibody-dependent cytotoxicity. CP therapy was previously used for the treatment of SARS and Middle East Respiratory syndrome [31,32]. Based on these observations, CP was suggested as a potential therapy to help rescue us from the COVID-19 pandemic [33]. Of note, CP is also associated with transfusion-related adverse effects such as chills, anaphylactic reaction, fever and generalized jaundice, as reported in a meta-analysis of the H5N1 pandemic [34].

CP transfusion in five critically ill COVID-19 patients with ARDS improved the clinical status of all the patients. However, the author was not able to conclude the efficacy of CP as it was a small sample study with no control subjects [35]. In addition, the subjects were also on antiviral therapy. A pilot study from ten severe COVID-19 cases showed that 200 ml of viral inactivated CP transfusion resulted in increased COVID-19 neutralizing antibodies and decreased the viral load 7 days post-treatment. The patients also observed decreases in pulmonary lesions post-treatment [36]. However, this study showed that only one out of ten critically ill patients showed some adverse effects in the form of facial red spots [36].

Intriguingly, neutralizing antibodies against the spike antigen of SARS-CoV-2 have previously shown detrimental acute lung injury in Chinese rhesus monkeys, despite clearing viral loads [37]. Similarly, SARS-CoV-2 deceased patients developed SARS-induced neutralizing antibodies much faster, which also correlated with acute lung injury that further suggests the role of neutralizing antibodies in inducing acute lung injury [38]. As the clinical study includes a small number of the test subject, the effectiveness of CP therapy is under further investigation in the form of a large number of randomized clinical trials. More recent randomized trials have further raised doubt as there was no clinical improvement in the CP treated and placebo group in severe COVID-19 cases [39,40].

### Tocilizumab

Reports have revealed increased levels of IL-6 in critically ill COVID-19 patients [41–43]. Hence, it was proposed that IL-6 blockade may be helpful in the amelioration of IL-6 induced immuno-pathology [44]. Tocilizumab (TMB) is a monoclonal antibody against the IL-6 receptor, which has been approved for the treatment of rheumatoid arthritis [45].

A study published with 15 test participants with confirmed COVID-19 revealed that TMB treatment significantly improved the inflammatory response of ten patients. However, two patients showed a detrimental response against the treatment, and three patients died [46]. The above clinical study advocated the role of TMB in dampening the cytokine storm, but it also stated the ineffectiveness of TMB in critically ill patients. On the other hand, TMB treatment of 25 critical COVID-19 patients, belonging to a different ethnic group, improved the mortality and clinical outcome of the disease because of a decreased inflammatory response. The study also documented minor adverse effects in 23 patients, most commonly anemia and a rise in alanine aminotransferase [47]. In another clinical study, 21 patients with a severe form of COVID-19 showed therapeutic efficacy of TMB treatment. Elevated IL-6 levels were diagnosed in all the patients before TMB treatment. Following TMB treatment, all the patients showed improvement in body temperature, while the majority of patients resulted in improved clinical symptoms such as C-reactive protein and lungs abnormalities [48]. Hence, it is quite relevant to judge the effectiveness of TMB based on the above observation. However, in a recently published randomized large sample-sized clinical trial, it was mentioned that TMB treatment results in a reduction in mortality [49], nevertheless it was a small improvement. In contrast, another randomized clinical trial with severe COVID-19 patients showed that TMB does not improve clinical status or lower mortality [50].

## Promising drugs for COVID-19 therapy

In this section, we have mentioned some promising drugs for COVID-19. These drugs are still in the preclinical and clinical trial stage for COVID-19 research, and as there is limited information and research on these drugs we tried to examine them more. Initials experiments with these drugs have shown promising results and hence in-depth studies may be anticipated shortly.

### Ivermectin

Ivermectin is an FDA-approved anti-filarial drug, which earlier has shown potent antiviral activity against HIV and Dengue virus [51]. Keeping its antiviral property in mind, Caly *et al.* tested the *in vitro* efficacy of ivermectin against SARS-CoV-2. The Vero/hSLAM cells were infected with SARS-CoV-2 and after 2 h post-infection these infected cells were treated with 5 μM ivermectin. The study revealed that ivermectin treatment resulted in a 93% reduction in the released virion and a 99.8% reduction in intracellular virus 24 h post-treatment. Furthermore, complete clearance of the virus was observed 72 h post-treatment [52]. Recently, a small clinical trial of ivermectin was published [53,54]. One study suggested that ivermectin can lower the viral titer in the treated groups [54] The most recent study also showed that ivermectin can contribute to viral decay, however, a significant difference was observed in patients with increased ivermectin in serum and not in individuals with low serum ivermectin [53]. This variability in serum ivermectin concentration could suggest polymorphism in genes regulating the bioavailability of the drug. Neither of the studies noticed any major adverse effects in the treatment group, which may be due to small enrolled test subjects. New, well-conducted, and sufficiently powered clinical trials are needed to judge the role of ivermectin in the treatment of COVID-19.

### EK1C4

EK1C4 is a lipopeptide derivative of coronavirus fusion inhibitor that inhibits the *in vitro* infection of live coronaviruses such as SARS-CoV-2, Middle East Respiratory syndrome coronavirus, human coronavirus (HCoV)-OC43, HCoV-229E and HCoV-NL63 [55]. The lipopeptide was able to block the infection of SARS-CoV-2 in a dose-dependent manner with IC_50_ of 36.5 nM. Furthermore, 0.5 h pretreatment of EK1C4 through the intra-nasal route before challenging the mice with human coronavirus, HCoV-OC43, resulted in a 100% survival rate [55]. With its low toxicity and peptidic nature, EK1C4 could be granted safe for humans. However, it will be too early to comment on the EK1C4 due to the preclinical nature of the study, and more evidence is required to test the efficacy and safety of EK1C4.

### Baricitinib

Baricitinib is an FDA-approved Janus Kinase (JAK) inhibitor for the treatment of rheumatoid arthritis. Additionally, baricitinib has also been shown to inhibits AP2-associated protein kinase 1, a critical regulator of Angiotensin-Converting Enzyme 2 receptor endocytosis. Hence, it was postulated that baricitinib could inhibit viral entry into the lungs [56]. As baricitinib is a potent inhibitor of JAK and therefore it may be also beneficial in subsiding too much inflammation in COVID-19 infected patients [56]. However, there is a risk associated with the use of baricitinib as it could also block JAK-mediated interferon production in COVD-19 patients, worsening the diseased state [57]. Therefore, based on its pharmacodynamic properties, clinical trials have been registered as NCT04320277 and NCT04358614 to further check the efficacy of baricitinib against COVID-19. In addition, a randomized double-blind placebo-control clinical trial was conducted across eight countries at 67 different sites, encompassing around 1000 test subjects [58]. The patient who received a combinational therapy of baricitinib and remdesivir recovered faster than compared to the control group.

## Variable responses to SARS-CoV-2 infection

In the current pandemic, we have observed variable host responses to SARS-CoV-2 infection. The most obvious reason could be the emergence of variants of concern in some countries [59,60] Furthermore, this could be also attributed to key human immune response determinants, age, sex, etc [61]. Variants of RIG-I could contribute to several viral infections as a result of the defective innate immune response [62]. Recently, it was reported that RNA viral sensors such as RIG-I protect against SARS-CoV-2 infection and defective RIG-I could exacerbate the disease [63]. Polymorphism in mitochondrial antiviral-signaling gene, downstream of RIG-I, results in a reduction of Type I interferon response [64], which is critical for viral protection. In addition, polymorphism in IL-6 -174G/C was associated with higher IL-6 production and severe pneumonia [65].

In addition to the presence of variants in the immune response, heterogenicity in drug metabolic enzymes and proteins could be the reason there is so much variable response to COVID-19 therapy [66]. Genetic polymorphism in some of the genes associated with drug metabolism have been reported. Chloroquine and HCQ drug are both metabolized by cytochrome P450 CYP2D6 [66]. Polymorphism in CYP2D6 can affect the HCQ metabolism and therefore the CYP2D6 genotype may be considered before HCQ treatment [67]. Similarly, CYP2D6 is also involved in remdesivir metabolism and functional variants may alter the pharmacodynamics of remdesivir [66]. In the case of lopinavir/ritonavir, the drugs are transported by ABC transporters (ABCB1 and ABCB2). Where ABCB1 polymorphism has a negligible effect on lopinavir/ritonavir, ABCB2 polymorphism can lead to accumulation of lopinavir/ritonavir in peripheral blood mononuclear cells of HIV patients, resulting in drug toxicity [68]. Overall, it is suggested that the polymorphism in proteins responsible for drug transport or metabolism can further regulate the drug pharmacokinetics and hence determine the clinical outcomes.

## Personalized therapy for COVID-19 mitigation?

**Figure 1.**
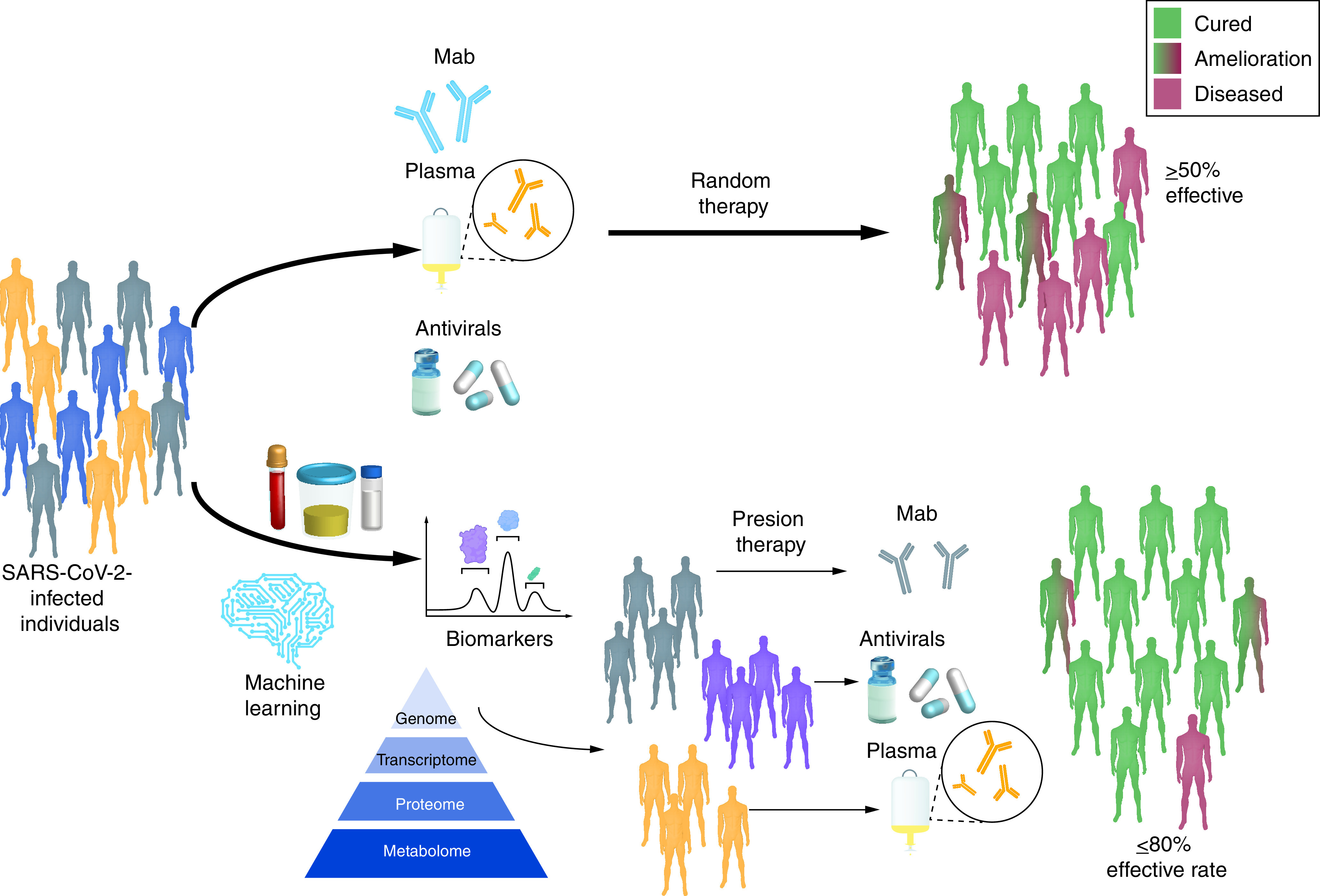
The landscape for personalized therapy in COVID-2019. The infected population that is segregated based on genotyping and critical immune signatures are subjected to different treatments such as anticytokine monoclonal antibodies treatment, plasma and antivirals. The optimized treatment accordingly can be helpful in a much better response to current therapy.

Personalized medicine is not a new approach, health workers and doctors have been relying on personalized medicine to cure cancer for the past few decades. Similarly, the use of personalized therapy is also being suggested in the time of COVID-19 [69,70]. As there is heterogeneity in the host response to SARS-CoV-2, a more precise and tailored treatment is required for favorable outcomes. It is further anticipated that identification of immune signatures in patients through wide genomic and proteomic analysis can be helpful in segregating the population, leading to more specific treatment and an improved response to the therapy (Figure 1).

## Personalized medicine in hospitalized patients

There are quite a few examples that recommend the implementation of personalized therapy in hospital settings. In the wake of determining the optimized treatment for COVID-19, different biomarkers have been studied for their correlation with the COVID-19 severity. Pulmonary inflammation index has helped predict the transition of mild to severe COVID-19. Combining pulmonary inflammation index with cytokine profiling and immune signatures can further help predict the severity of COVID-19 disease and optimized treatment [71]. Recently, Endothelial Activation and Stress Index was suggested to predict the outcome of COVID-19 severity. This index predicts endothelial dysfunction and relies on lactate dehydrogenase (LDH), creatinine and platelet counts. The scores are correlated with typical markers for COVID-19 such as increased serum and inflammatory markers [72]. Finally, it is suggested that the identification of critical biomarkers can predict the severity of COVID-19 [63,72] and could pave the way for personalized medicine.

In a small study, the COVID-19 patients were distinguished based on different complications such as IL-6 mediated ARDS, inflammatory response, co-infection, immune dysfunction due to C-reactive proteins, and hyper-ferritinemia. IL-6 and IL-1 blockade were helpful in patients with ARDS, patients with co-infection undergo appropriated microbial culture for antibiotic treatment. Overall, the study showed that the personalized approach was beneficial in improving conditions in about 94% of patients as compared with 60% [73]. A study published in The Lancet advocated the screening for hyper inflammation in patients and subsequently treated with immunosuppressive drugs could be beneficial in reducing the mortality caused as a result of acute lung injury [74]. In a retrospective small study conducted to test the therapeutic potential of TMB, the authors highlighted the limitations of the study such as the lack of checking the levels of IL-6 before and after TMB treatment, which is critical for gaining insight on the immuno-modulating effects of TMB [47]. Nevertheless, the prior knowledge of immune signatures in the infected patients can provide a better prognosis and critical insights for determining the roadmap for personalized medicine.

## Can we adopt personalized medicine in a pandemic?

Now the question arises, can a personalized therapy approach contain this pandemic? Despite developing advanced therapeutic techniques together with artificial intelligence, we are still not able to establish personalized medicine in our healthcare system. The latter is reflected by the mild to severe side effects in several clinical trials (Table 1). These side effects could be due to genetic variability between different individuals and hence one therapy may not be suitable for all. Taking these points into consideration, we can not rule out the importance of personalized therapy that could help dampen the current pandemic. Furthermore, heterogeneity in COVID-19 symptoms between individuals, due to different genetic factors, age, sex, race and ethnicity, the response to COVID-19 therapy is variable. Additionally, the emergence of new SARS-CoV-2 variants may compromise the current vaccine drive [75]. Hence now is the time to shift our healthcare system to a more focused approach. Due to the advancement in the biomedical field, now it is not impossible to incorporate the use of precision medicine in our medical practice. However, the only shortcoming of personalized medicine is the time required for the collection and analysis of data. Nevertheless, this can be overcome by the use of more sophisticated diagnostic tools with superior sensitivity. In addition, using artificial intelligence we will be able to predict the optimum drug and doses required for a patient for a better effective outcome [76].

## Conclusion

The emergence of COVID-19 has unequivocally taken the world by storm, leading to unprecedented loss of lives, financial challenges, mental and physical distress. Apart from the vaccine, there is no specific effective therapy for COVID-19. Most of the drugs discussed in this review are repurposed drugs, however, these drugs have shown inconsistent results and sometimes severe to mild side effects. Drug failure could be due to heterogeneity in SARS-CoV-2 and hosts such as sex, age, and other biological factors. Nevertheless, the role of genetic polymorphism in genes related to drug metabolism and bioavailability can also be suggested. To this end, we suggest a more precise way for COVID-19 management that may require identifying risk factors in mild or severely diseased patients. In addition, biomarker-based identification could help in diagnosing the severity of the disease and hence could help speed up personalized care.

## Future perspective

Due to the unfamiliar nature of SARS-CoV-2, clinicians have been skewed toward the use of repurposed drugs. Although the safety of these drugs has been pre-established, there are some cases of clinical repercussions that need to be addressed properly. Of note, the latter can be addressed by proper dosing of the repurposed drugs as these drugs were previously optimized for other diseases [77]. As the dynamics of the SARS-CoV-2 infection is still evolving, we need to critically examine the pharmacodynamics and toxicity of drugs against COVID-19 by conducting a large number of randomized clinical trials. A comprehensive evaluation of potent drug candidates under clinical trials may be helpful in the generation of a roadmap to deal with the current COVID-19 pandemic. More importantly, it is time to incorporate a personalized approach or individualized medicine in our healthcare system. The latter can be achieved easily through a genomic, proteomic and metabolomic study, and together with a computational approach such as machine learning (Figure 1). Furthermore, it is now possible to predict drug effectiveness and drug response in an individual [78]. In addition, a personalized approach will further lower the risk of adverse events following any therapy. We certainly believe that COVID-19 has propelled us to move towards personalized medicine in a way that will not only help overcome the current pandemic but also help overcome other emerging pathogens.

**Executive summary**

- Current COVID-19 therapy is still in search of an optimum drug. Clinical trials with repurposed drugs sometimes reported toxicity and severe side effects. As COVID-19 is a pandemic, these side effects and drug toxicity may be observable due to the involvement of heterogeneity in test populations such as race, sex, age and polymorphism in key determinants.
- These side effects can be substantially controlled using biomarker-based personalized therapy. COVID-19 patients have been diagnosed with a variety of immune signatures that help the patients to be classified with mild or severe COVID-19. Patients with a hyperimmune response during early infection benefited from anti-inflammatory drugs rather than antivirals. On the other hand, the increase in IL-6 in serum is generally observed in COVID-19 patients, and treatment with IL-6 receptor monoclonal antibody, tocilizumab, resulted in improved clinical symptoms. These few observations suggest that a personalized approach may be critical for COVID-19 management. However, still, large-scale adoption of a personalized medicine approach is missing from the healthcare system.
- Due to recent advancements in biomedical sciences, with the integration of artificial intelligence and machine learning, it is now possible to easily diagnose the severity of COVID-19. Hence, the implementation of personalized medicine may prove beneficial in containing the current pandemic.
